# The management of solitary fibrous tumor: a single center's experience

**DOI:** 10.3389/fsurg.2026.1874427

**Published:** 2026-08-07

**Authors:** Han-Hung Lee, Chao-Kun Chen, Yao Fong, Ying-Chieh Su

**Affiliations:** 1 Department of Chest Surgery, Tung’s Taichung MetroHarbor Hospital, Taichung, Taiwan; 2 Division of Thoracic Surgery, Department of Surgery, Chi Mei Medical Center, Tainan City, Taiwan

**Keywords:** pleural neoplasm, recurrence, risk stratification, solitary fibrous tumor (SFT), video-assisted thoracic surgery (VATS)

## Abstract

**Background:**

Solitary fibrous tumor (SFT) is a rare mesenchymal neoplasm, with about 30% occurring in the intra-thoracic area. This study describes the clinical characteristics and management strategies for SFT in a single center.

**Methods:**

A retrospective review was conducted on patients with pleural SFT who underwent surgery at our institution from January 2005 to August 2023. Data included demographics, tumor features, surgical details, pathology, and follow-up outcomes.

**Results:**

Seventeen patients underwent 19 surgeries. The median age was 57 years, and 41% of patients were male. Right-sided tumors accounted for 63% of cases, and 26% of tumors exceeded 10 cm. VATS was used in 68% of procedures. Blood loss varied widely from 50 to 8,600 mL, with a median hospital stay of 6 days. Risk classification included 9 low-risk, 7 intermediate-risk, and 3 high-risk surgical cases. Survival status was available for 13 patients; 12 were alive, and 1 had died from unrelated lung cancer.

**Conclusion:**

This single-center experience suggests that complete resection remains central to the management of pleural SFT, and VATS may be feasible in selected cases. Preoperative planning may be useful for large or hypervascular tumors, and long-term follow-up should be considered because recurrence with pathological progression may occur. These findings should be interpreted cautiously given the small sample size and retrospective design.

## Background

Solitary fibrous tumor (SFT) is a rare mesenchymal neoplasm that predominantly arises from the pleura but can occur in various other anatomical sites (1). According to the data from The Surveillance of Rare Cancers in Europe (RARECARE), an analysis of a large population treated across 89 European centers shows that the incidence of SFT is less than 0.1 per 100,000 person-years (2). Although SFTs are generally benign, a subset can exhibit aggressive behavior, including significant local recurrence, invasive growth, and, in rare cases, metastasis (3). The clinical presentation of SFTs varies widely, often complicating diagnosis and management (4). Key factors influencing the clinical course include tumor size, location, and histopathological characteristics, such as mitotic activity and necrosis (5).

Recent advances in imaging and histopathological techniques have improved our understanding of SFTs, facilitating more accurate risk stratification and individualized treatment planning (6, 7). The cornerstone of SFT management remains surgical resection, aiming for complete tumor removal with clear margins (4). However, the potential for significant intra-operative bleeding and other complications necessitates meticulous preoperative planning and intraoperative management strategies (8). In particular, the role of preoperative embolization in minimizing operative blood loss has gained prominence, alongside the evolving application of perioperative radiotherapy in select high-risk cases (9).

Nevertheless, gaps remain in current knowledge. Most published large series focus on overall outcomes, but rarely emphasize uncommon clinical scenarios such as acute massive hemothorax, repeated recurrences with pathological progression from benign to malignant features, or management of highly vascularized giant tumors requiring combined strategies (e.g., preoperative transarterial embolization and intraoperative argon plasma coagulation) (10–12). These situations pose substantial challenges for thoracic surgeons and are underrepresented in the literature. This study therefore aims to provide a comprehensive review of the clinical characteristics, treatment strategies, and outcomes of SFTs at our institution. A secondary aim was to highlight uncommon management challenges through selected illustrative cases. By analyzing a series of cases treated at our center, we seek to elucidate factors influencing recurrence, surgical safety, and survival, thereby contributing novel insights to the broader understanding and management of this complex tumor entity.

## Methods

### Study design and patient selection

This retrospective study reviewed the medical records of patients diagnosed with pleural solitary fibrous tumor (SFT) who underwent surgical intervention at Chi Mei Medical Center between January 2005 and August 2023. Patients with pathologically confirmed pleural SFT were included in the analysis.

### Data collection

Clinical data were collected from medical charts, including demographic characteristics, tumor features, surgical procedures, intraoperative blood loss, operative time, length of hospital stay, and pathological findings. Pathological variables included immunohistochemical staining results, mitotic activity, and the presence of necrosis. Follow-up information, including tumor recurrence and survival outcomes, was also reviewed and analyzed. The Demicco risk scoring system is determined according to patient age, tumor size, and mitotic count (13).

## Results

Seventeen patients underwent 19 surgeries. The cohort had a median age of 57 years (IQR: 47.0–69.0), with 7 (41%) being male. Right-sided lesions were present in 12 (63%) cases, and 5 (26%) tumors exceeded 10 cm in size. Video-assisted thoracic surgery (VATS) was employed in 13 (68%) procedures. Blood loss varied from 50 to 8,600 mL, with a median hospital stay of 6 days. Available cohort-level outcomes are summarized in Table 1. Risk classification was available for all 19 surgical cases, including 9 low-risk, 7 intermediate-risk, and 3 high-risk cases. Survival status was available for 13 patients, among whom 12 were alive and 1 had died from unrelated stage IV lung cancer. However, follow-up duration, margin status, and recurrence status were not uniformly available across the entire cohort and therefore could not be analyzed systematically.

**Table 1:** Patients’ demographics and clinical characteristics.

Variables	Statistics
Age, years	57.0 (47.0—69.0)
Sex^a^	
Female	10 (58.8%)
Male	7 (41.2%)
Side	
Left	7 (36.8%)
Right	12 (63.2%)
Tumor size (length, cm)	6.0 (3.0 - 11.0)
Tumor size >10 cm	5 (26.3%)
VATS	13 (68.4%)
OP time (min)	110 (75, 220)
Blood loss (mL)	50 (50, 250)
Days of admission	6 (5, 10)
Necrosis	6 (31.6%)
Risk score	
High (5-6)	3 (15.8%)
Inter-mediate (3-4)	7 (36.8%)
Low (0-2)	9 (47.4%)
Survival status^a^	
Expired	1 (7.7%)
Alive	12 (92.3%)
Missing	4

OP time, operation time; VATS, video-assisted thoracic surgery.

a Count as 17 patients.

Here we highlight three representative cases to demonstrate the clinical presentation, surgical management, and outcomes of SFT.

## Representative cases

### Case 1

**Figure 1:**
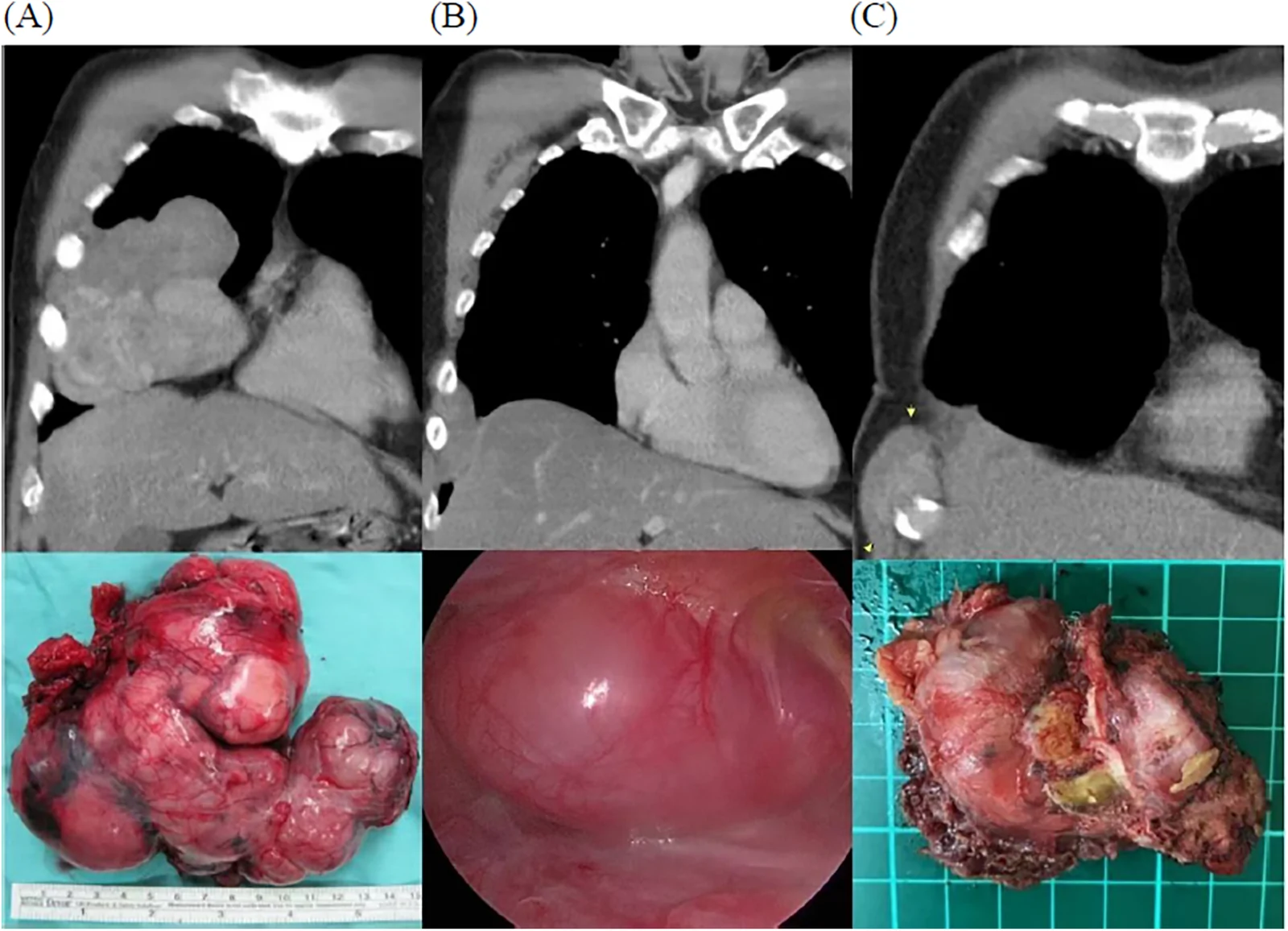
From left to right: **(A)** the preoperative CT scan (top) for the en-bloc resection of a chest wall tumor and right middle lobe (RML) wedge resection, along with the resected specimen; **(B)** the CT scan before the second surgery (video-assisted thoracoscopic surgery, vATS) for excision of a pleural-based tumor, accompanied by intraoperative vATS photos; and **(C)** the CT scan prior to the third surgery (chest wall resection and reconstruction), along with the excised specimen.

A 52-year-old male with no significant medical history initially presented with persistent productive cough, leading to the discovery of a SFT through a CT-guided biopsy. Following this diagnosis, he underwent a comprehensive intervention, including a 375-minute en-bloc resection of a chest wall tumor and wedge resection of the right middle lobe (RML), due to invasion into the right lung and adherence to the 5th and 6th ribs. This procedure resulted in minimal blood loss of 500 mL. Notably, the pathological examination revealed less than 2 mitotic figures per 10 high-power fields, along with positivity for CD34 and a low proliferation index in immunohistochemical studies. The patient was discharged on postoperative day 7, with a meticulous follow-up plan in place. **(**Figure 1A**).**

After a 74-month remission period, the patient experienced an asymptomatic recurrence near the previous operative site. Despite being symptom-free, he reported weight loss over three months. Subsequently, he underwent video-assisted thoracoscopic surgery (VATS), lasting 125 minutes, for the excision of a pleural-based tumor, which measured 6.63 cm. The procedure resulted in minimal blood loss of 250 mL. Pathological analysis revealed an increase in mitotic figures to 2 per 10 high-power fields, along with CD34 positivity and negative staining for TTF1, AE1/AE3, and EMA. Discharged on postoperative day 7, he continued with regular follow-up for any potential recurrence or complications. **(**Figure 1B**).**

Following a recurrence of the nodule 37 months later, the patient underwent a 220-minute chest wall resection and reconstruction. Minimal blood loss of 100 mL was noted during this procedure. Pathological examination revealed an increase in mitotic figures to 4 per 10 high-power fields, with no evidence of tumor necrosis. Immunohistochemical analysis showed diffuse positivity for STAT6. Discharged on postoperative day 9, the patient remains under regular surveillance, showing no signs of disease recurrence thus far. **(**Figure 1C**).**

Figure 1 presents CT images, intraoperative VATS photos, and photos of the excised specimens from the abovementioned procedures.

### Case 2

**Figure 2:**
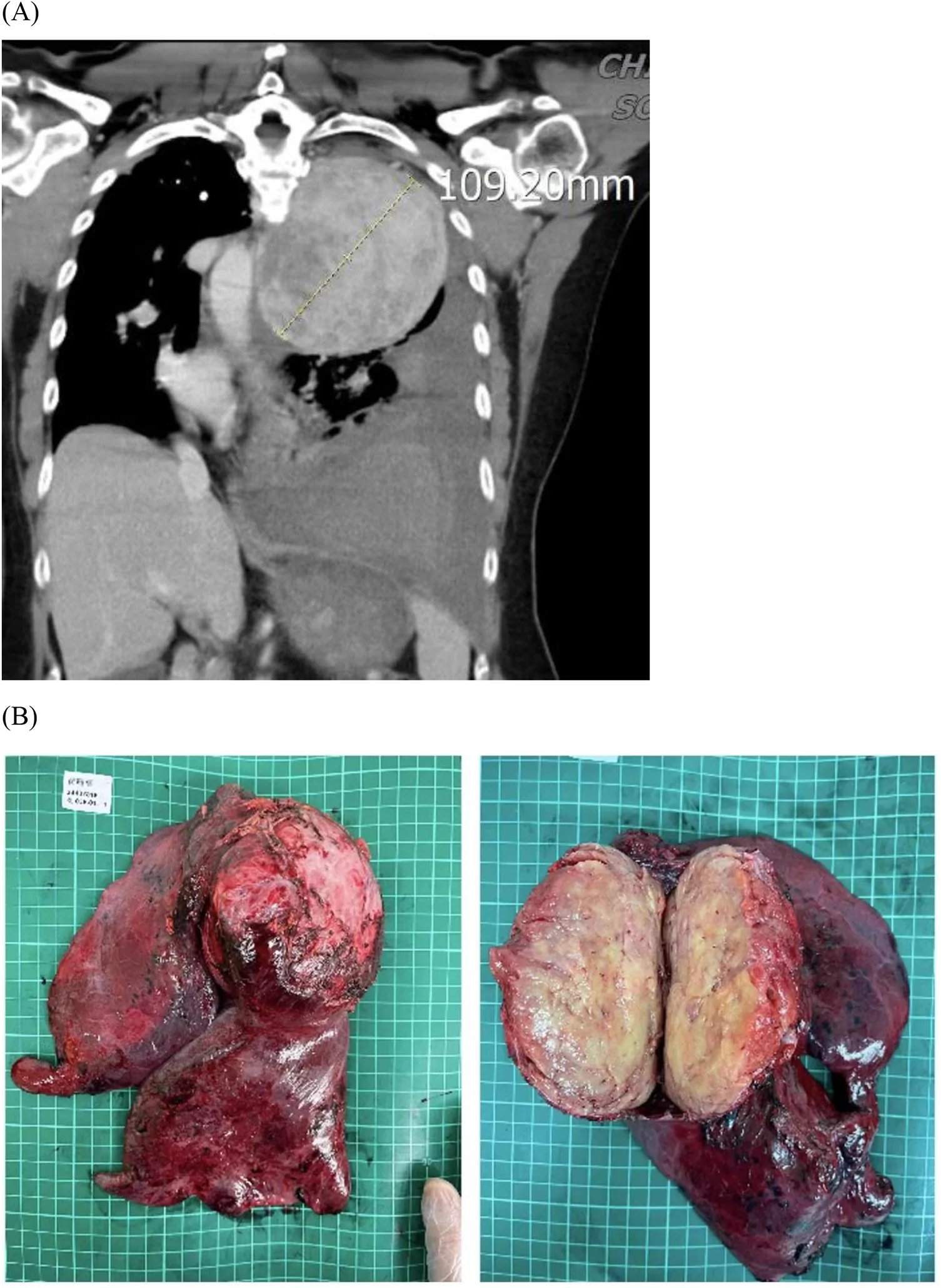
**(A)** chest radiogram findings showed a massive left pleural cavity, **(B)** A left pneumonectomy was performed via sternotomy and thoracotomy.

A 75-year-old female, without significant medical history, presented with progressive dyspnea over three days. Imaging revealed a complete white-out of the left pleural cavity on chest radiogram **(**Figure 2A**)**. Pigtail insertion resulted in bloody effusion, with a rapid drop in hemoglobin from 11.7 to 8.1 within one day. VATS removal of a 550 mL blood clot was performed four days after admission, revealing a large left upper lobe lung tumor with adhesion to the chest wall **(**Figure 2B**)**. No pleural seeding was observed, and tumor markers were within the normal range. Further investigations ruled out brain metastasis but indicated an intermediate probability of bony metastasis at T11. CT-guided biopsy showed uniform fibroblastic spindle cells with few mitoses and no necrosis. Immunohistochemical stains were positive for CD34 and STAT-6 but negative for AE1/AE3 and calretinin. Following diagnosis, the patient was discharged on day 13 but later underwent left pneumonectomy via sternotomy and thoracotomy due to intermediate-risk features. The procedure, lasting 537 minutes with significant blood loss (8,600 mL), resulted in tumor-free margins. Risk assessment categorized the tumor as intermediate risk, and the patient was discharged on day 13 post-surgery. The patient remains under regular surveillance, showing no signs of disease recurrence thus far.

### Case 3

**Figure 3:**
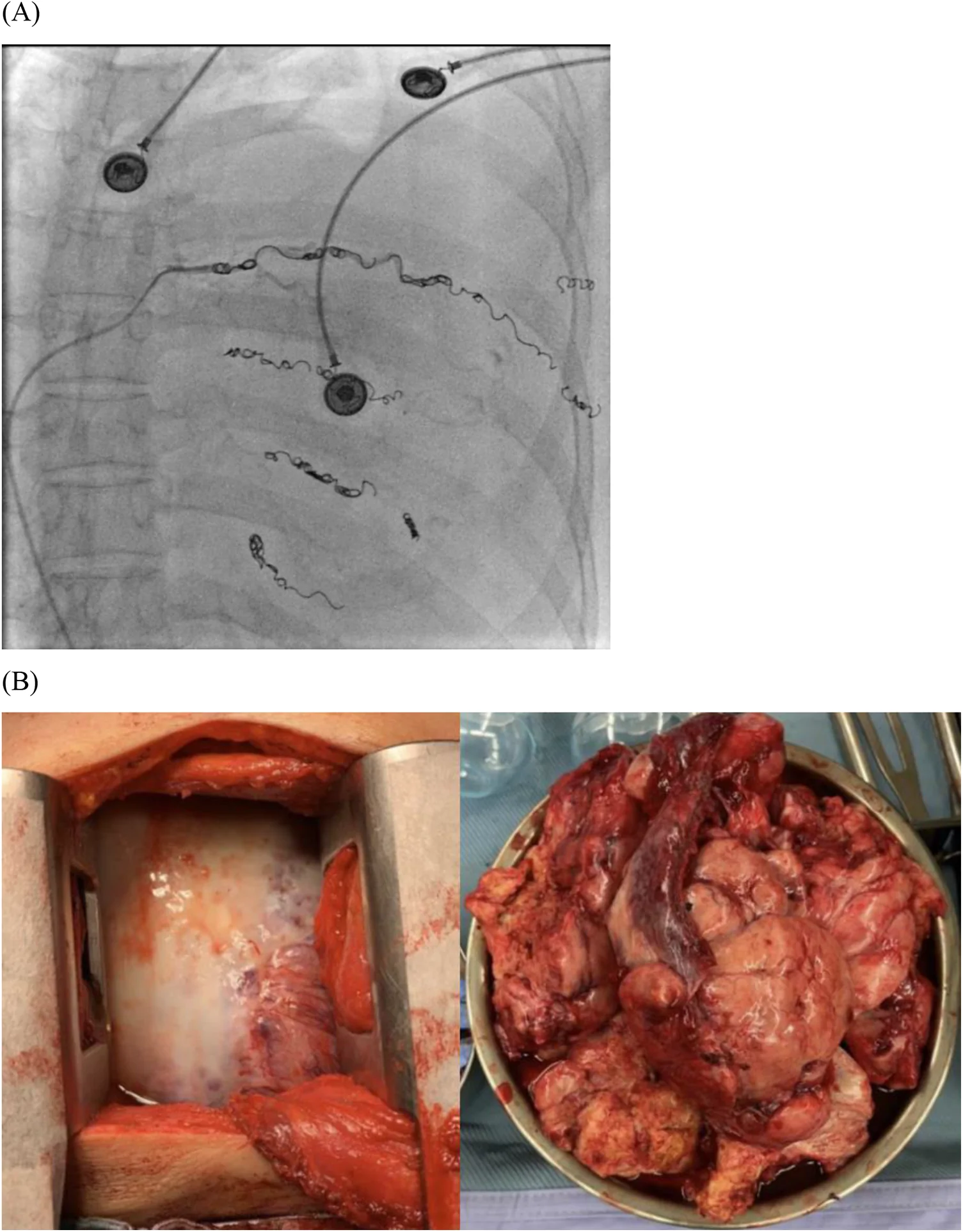
**(A)** Pre-operative TAE was performed for a large hypervascular mass supplied by the left internal thoracic, lateral thoracic, and T7-T9 arteries, achieving complete tumor devascularization. **(B)** On the same day after TAE, a 1900g pleural tumor was resected, with diffuse chest wall oozing controlled by argon plasma coagulation (APC).

A 56-year-old female, without significant medical history, presented with progressive dyspnea over 1-2 months. Chest sonogram revealed minimal yellowish cloudy pleural effusion, precluding pigtail insertion. A sono-guided biopsy performed three days after admission revealed a spindle cell tumor with bland cells, positive for STAT6 and CD34. She was readmitted one month later for preoperative transarterial embolization (TAE) to devascularize a huge hypervascular mass lesion supplied by multiple arteries **(**Figure 3A**)**. Following TAE, complete devascularization was achieved, enabling the resection of the pleural tumor on the same day. The procedure lasted 436 minutes with significant blood loss (4,800 mL) and a tumor weight of 1900 gm. Diffuse oozing of the chest wall was controlled by argon plasma coagulation (APC) **(**Figure 3B**)**. Pathological examination revealed tumor-free margins and immunohistochemical staining positive for STAT6, CD34, and CD99. Risk assessment categorized the tumor as high risk due to age (> 55 y/o), tumor size (> 15 cm), mitotic count (5/10 HPF), and tumor necrosis (> 10%). The patient was discharged on day 14 post-surgery. The patient remains under regular surveillance, showing no signs of disease recurrence thus far.

## Discussion

In the present cohort, most patients underwent complete surgical resection, and VATS was feasible in selected cases. Our tumor characteristics, including tumor size and malignant potential, were generally consistent with previously reported data (9). However, we identified several notable cohort-level findings, including a relatively high necrosis rate (31.6%), highly variable intraoperative blood loss with rare extreme bleeding events up to 8,600 mL, and one case of recurrence with pathological progression.

These findings support the importance of complete resection, which has been widely regarded as the cornerstone of treatment for pleural SFTs (4). The recurrent case in our cohort also reinforces the need for long-term surveillance, as previous studies have reported malignant transformation in initially benign recurrent SFTs (4, 14).

Based on our experience, selected large or hypervascular tumors may require additional preoperative planning, including consideration of embolization, although this should not be interpreted as routine practice. Overall, our findings support a management strategy centered on complete resection, individualized perioperative planning, and structured postoperative follow-up, while the conclusions should remain cautious because of the limited sample size.

In our cohort, immunohistochemical findings, particularly CD34 and STAT6 positivity when available, supported the pathological diagnosis of SFT. In the broader literature, immunopathological markers also play a role in distinguishing benign from malignant SFTs. Benign tumors typically stain positive for CD34, Bcl-2, CD99, STAT6 (nuclear), and GRIA2, while showing negativity for pan-cytokeratin, EMA, SMA, Desmin, CD31, and S100. Conversely, malignant tumors exhibit positivity for p53, BCL-2, CD99, STAT6 (nuclear), and GRIA2, with variable expression of CD34, cytokeratin, and beta-catenin (15, 16). Beyond immunohistochemical staining patterns, differentiation can also be achieved through clinical presentation, imaging characteristics, and histopathological features. This multimodal assessment aids in accurately categorizing SFTs and guiding appropriate treatment strategies, ultimately improving patient management and outcomes (17).

In our hospital, risk stratification of SFTs is conducted using the Demicco system, which considers age, tumor size, and mitotic count (13). This system was applied when the relevant variables were available in this retrospective cohort. However, various parameters have been adopted across different studies to assess the risk of malignancy and metastasis. De Perrot's classification focuses on pathological features such as benign or malignant behavior and the presence of multiple metastases (18), while Tapias’ system considers factors like tumor origin, morphology, size, and histological characteristics (19). Boddaert and colleagues found Tapias’ system to have the highest sensitivity and specificity for risk assessment. Additionally, studies have highlighted the significance of areas of dedifferentiation within SFTs, with significantly higher rates of metastasis and disease-specific mortality observed in such cases compared to histologically benign tumors (20, 21). This underscores the importance of utilizing comprehensive risk stratification methods to guide clinical decision-making and optimize patient management. In the present study, pathological variables such as mitotic activity and necrosis were recorded when reported, but were not uniformly available across all cases, reflecting the inherent limitations of retrospective data.

In our cohort, APC was used as an adjunctive hemostatic technique in one complex hypervascular case. APC has been reported as a useful method for controlling bleeding in selected surgical or endoscopic settings (22). However, evidence specific to APC use in SFT surgery remains limited. Therefore, this finding should be interpreted cautiously as case-level experience rather than systematic evidence.

With respect to surgical procedures, preoperative embolization of feeding arteries has been recognized as a potential strategy for reducing intraoperative blood loss, particularly in cases of large SFTs exceeding 20 cm. Studies have highlighted the possible efficacy of this approach in facilitating safer tumor resection. Preoperative angiography and embolization can help decrease intraoperative bleeding by identifying and managing major feeding vessels (14, 23, 24). However, embolization is not without limitations, and complications such as medullary ischemia may occur. Timing is crucial, with surgery ideally performed within 24 hours of embolization to optimize effectiveness (25). In our hospital, despite preoperative transarterial embolization (TAE), venous bleeding remained a challenge during surgery. Hemostasis was successfully achieved using argon plasma coagulation (APC), illustrating its role as a salvage or adjunctive technique rather than definitive proof of efficacy in this setting.

Perioperative radiotherapy, chemotherapy, and targeted therapies were not systematically evaluated in our cohort. Therefore, the following discussion is intended only as literature-based context for selected high-risk or unresectable SFTs. Perioperative radiotherapy may improve local control in selected high-risk SFTs, particularly those with high mitotic activity or unclear surgical margins, although its effect on overall survival remains uncertain (17, 26). Postoperative radiotherapy may benefit cases of mediastinal SFTs with external invasion and may facilitate subsequent resection in selected cases (27). However, the impact of perioperative radiotherapy on overall survival remains uncertain. Chemotherapy has limited efficacy, and targeted therapies such as pazopanib or apatinib remain investigational in selected cases. Because these treatments were not evaluated in our cohort, they should be interpreted cautiously and individualized according to tumor behavior, surgical margins, and patient factors.

### Limitations

This study has several limitations. Firstly, the small sample size significantly limits the generalizability of the findings and hinders the ability to draw definitive conclusions, particularly regarding survival differences between patients in different risk categories. Second, due to the retrospective nature of this study, several baseline clinical parameters—such as performance status, margin status, local invasion, baseline metastasis, and mitotic rate—were not uniformly available across all cases, and risk stratification was therefore applied only when the relevant variables were documented, which limits the robustness of prognostic evaluation. The follow-up period was heterogeneous and may not have been long enough to fully capture long-term outcomes, including late recurrences or complications. Although SFTs are prone to local recurrence, cohort-level recurrence rates and time-to-event outcomes could not be systematically derived due to variable follow-up imaging strategies. Accordingly, the outcome analyses in this study should be interpreted as primarily descriptive. No survival analysis was performed, and complete survival status was not available for all patients, further limiting outcome interpretation. We also lacked data on perioperative radiotherapy or chemotherapy, limiting our ability to evaluate the full range of treatment options and to identify the ideal candidates for perioperative therapy beyond surgery. Finally, as the findings are based on a single center's experience, they may not reflect broader clinical practice. Further studies with larger, multicenter cohorts, standardized follow-up protocols, and more complete clinicopathological data, such as recurrence and margin status, are required to validate these results and explore additional treatment strategies.

## Conclusion

Surgical treatment is widely regarded as the standard for managing SFTs, with complete, margin-free resection being a fundamental principle. When necessary, extensive resection, including chest wall resection and reconstruction, may be required to achieve thorough tumor removal and to minimize the theoretical risk of contact tumor dissemination. VATS appears to be a feasible option in appropriate cases, particularly when combined with careful pre-operative planning. This includes risk stratification and, in selected cases, pre-operative embolization within 24 hours before surgery as a potential adjunct to reduce intra-operative bleeding. Effective hemostasis, using techniques like APC, may serve as an adjunctive measure for controlling venous bleeding based on limited case-level experience. Despite successful resection, recurrence remains a possibility, especially in cases with malignant potential as indicated by pathological findings. Therefore, the use of risk stratification tools and vigilant post-operative surveillance should be considered important components of clinical management. Overall, treatment strategies should be individualized based on pathological and clinical assessments, and the conclusions drawn from this single-center experience should be interpreted with caution. This study findings should be interpreted as exploratory. Further research is needed to refine these approaches and explore additional therapeutic options, including the potential role of targeted therapies.

## Data Availability

The original contributions presented in the study are included in the article/Supplementary Material, further inquiries can be directed to the corresponding author/s.
